# *MYC* Gene Rearrangements Are Closely Associated with Poor Survival of Diffuse Large B Cell Lymphoma with Hepatitis B Virus Infection

**DOI:** 10.1155/2017/1967648

**Published:** 2017-10-25

**Authors:** Zhihe Liu, Siyun Li, Wei Guo, Yinping Wang, Ming Wang, Ou Bai

**Affiliations:** ^1^Department of Hematology, The First Hospital of Jilin University, Changchun 130021, China; ^2^Department of Pediatrics, Women and Children's Hospital of Qingdao University, Qingdao 266000, China; ^3^Department of Pathology Diagnosis Center, The First Hospital of Jilin University, Changchun 130021, China

## Abstract

The aim of this study was to identify clinical adverse prognostic factors affecting overall survival (OS) of diffuse large B cell (DLBCL) patients with hepatitis B virus (HBV) infection. In this study, 30 DLBCL patients with HBV infection and 51 DLBCL patients with HBV-free were reviewed retrospectively. As of July 2016, the median follow-up period was 26.4 months (3.0~65.0 months). The median OS of patients in HBV infection group was 38.6 months, while that of patients in HBV-free group was not reached (*P* = 0.042); the median progression-free survival (PFS) of patients in HBV infection group was worse than that in HBV-free group, 18.5 months and 38.5 months (*P* = 0.118), respectively. The rate of* MYC* and* BCL2* gene rearrangements in HBV infection group was significantly higher than that in HBV-free group, 20.0% versus 3.9% (*P* = 0.019) and 23.3% versus 5.9% (*P* = 0.021), respectively. Multivariable analysis indicated that IPI (*P* = 0.002), chemotherapy regimens (*P* = 0.017), and* MYC* gene rearrangements (*P* = 0.004) were independent adverse prognostic factors for all DLBCL patients in this study. Results demonstrated that the poor survival of DLBCL patients with HBV infection was closely involved in chemotherapy regimens, IPI, and* MYC* gene rearrangements.

## 1. Introduction

HBV infection remains a serious public health problem. It is reported that there are nearly 2 billion people currently suffering from HBV infection, and some 360 million are believed to be infected with chronic HBV infection worldwide, which includes 93 million people in China [1–3]. Therefore, China currently is still a region where HBV is endemic. Furthermore, there are as high as 300,000 deaths per year caused by HBV-related diseases [4].

To the best of our knowledge, the pathogenesis of many diseases is closely associated with HBV infection. At present, it has already been confirmed that HBV infection can remarkably increase the incidence of lymphomas, especially DLBCL [5–7]. What is more, some researchers discover that the survival of DLBCL patients with HBV infection is poor, compared to those DLBCL patients without HBV infection [8, 9]. But to date, there are few articles systematically investigating clinical adverse prognostic factors of DLBCL patients with HBV infection. Therefore, the purpose of this study is to explore clinical adverse prognostic factors of DLBCL patients with HBV infection.

## 2. Materials and Methods

### 2.1. Patient Selection

A total of 201 patients initially diagnosed with DLBCL-NOS between January 1, 2011, and December 31, 2015, in the First Hospital of Jilin University were retrospectively reviewed. Of 201 patients, 182 patients had complete clinical information and received the first-line chemotherapy, such as R-CHOP, CHOP, and CHOP-like regimens. Patients with DLBCL transforming from low-grade lymphomas and those with primary cutaneous and primary central nervous system DLBCL, Epstein-Barr virus positive (EBV^+^) DLBCL, and hepatitis c virus infection (HCV^+^) DLBCL were excluded, and 81 patients were recruited in this study finally.

We retrospectively reviewed the results of HBV infection of all patients in this present study from our department of laboratory. Based on the results of HBV serum test, 30 patients were assigned to HBV infection group as the hepatitis B surface antigen was positive; 51 patients were assigned to HBV-free group (1, all HBV serum markers were negative; 2, HBV surface antibody positive and other serum markers negative should also be considered as HBV-free since some patients might receive HBV vaccine). All patients' diagnoses were in line with the lymphatic hematopoietic system malignant tumor classification standard (WHO, 2008) [10], and all patients' clinical stages were in accordance with the Ann Arbor staging system [11]. The study was conducted in accordance with the Helsinki Declaration, and the protocol had been approved by the ethics review committee of the First Hospital of Jilin University. Written informed consent was obtained from the patients or their legal guardians before carrying out the study.

### 2.2. Prognostic Factors

Prognostic factors included age, sex, clinical stage, molecular subtype (GCB and non-GCB), IPI, Ki-67,* MYC* gene rearrangements,* BCL2* gene rearrangements, and chemotherapy regimens. The GCB, non-GCB, and Ki-67 were examined by immunohistochemistry, which were routinely detected in Pathology Diagnosis Center. The other information, including age, sex, clinical stage, IPI, and chemotherapy regimens, was retrospectively collected in the database of the First Hospital of Jilin University. The immunohistochemistry results were evaluated blindly by two hematopathologists based on the current World Health Organization criteria and Hans algorithm, respectively [10, 12].

### 2.3. I-FISH for MYC and BCL2 Gene

The gene rearrangements of* MYC* and* BCL2* were detected by interphase fluorescence in situ hybridization (I-FISH) on formalin-fixed and paraffin-embedded (FFPE) lymphoma samples of 81 DLBCL patients using* MYC* and* BCL2* gene dual-color, break-apart probes (Vysis, Abbott Molecular, USA); more than 100 nuclei were examined for each probe whenever possible. 20 cases of patients with necrotic lymph node inflammation were selected as well to estimate the cutoff value of* MYC* and* BCL2* gene rearrangements. The cutoff value for the dual-color break-apart arrangement probes was established by evaluating the split signal distribution in samples of reactive lymphoid tissues, calculating the mean number of split signals plus 3 times the standard deviation; the cutoffs value for* MYC* and* BCL2* gene rearrangements was 8.9% and 9.8%, respectively.

### 2.4. Treatments

Before receiving chemotherapy, real-time quantitative polymerase chain reaction (RT-PCR) was used to test the HBV-DNA copy number. For patients with HBV-DNA copy number more than 1000 IU/ml, they did not receive chemotherapy until their HBV-DNA copy number was less than 1000 IU/ml after antivirus therapy (Lamivudine or Entecavir). In this study, all patients in HBV infection group received antivirus prophylaxis from initial chemotherapy to at least three months after completion of last chemotherapy for preventing HBV reactivation.

All patients achieved 2–8 cycles of first-line treatment, such as R-CHOP, CHOP, and R-CHOP/CHOP-like chemotherapy; for patients with progressive disease after first-line chemotherapy, they received second-line chemotherapy, for example, ICE, Gmox, GDP, ESHAP, DICE, and DHAP regimens. In order to monitor HBV reactivation, HBV-DNA copy number of patients in HBV infection group was performed before each course of chemotherapy.

### 2.5. Survival

OS was defined from diagnosis to death or last follow-up. PFS was defined from diagnosis to disease progression, death, or last follow-up.

### 2.6. Statistics

SPSS 20.0 software was used for the statistical analysis, and OS was analyzed using the Kaplan-Meier method. Single variable and multivariable analyses were performed by Cox regression analysis. General information and clinical efficacy were compared by the *X*^2^ test. *P* value ≤ 0.05 was regarded as statistical significance.

## 3. Results

### 3.1. Base Information

In this cohort, the prevalence of HBV infection among these DLBCL patients was 16.5% (30/182). In HBV infection group, the median duration time for antivirus prophylaxis was 8 months (5–13 months); out of these 30 DLBCL patients with HBV infection, 70.0% patients had Lamivudine therapy from initial chemotherapy to at least three months after completion of last chemotherapy, and 30.0% received Entecavir therapy; unfortunately, there were still three patients suffering from HBV reactivation during chemotherapy. Of these three patients, two received rituximab-containing chemotherapy and one adopted chemotherapy without rituximab, the rate of HBV reactivation was 10.0% (3/30). The first patient had HBV reactivation after the fifth cycle of rituximab-containing chemotherapy combined with Lamivudine therapy; the second patient contracted HBV reactivation after the third cycle of chemotherapy without rituximab combined with Lamivudine therapy; the last patient suffered from HBV reactivation after the second cycle of rituximab-containing chemotherapy combined with Lamivudine therapy. These three patients discontinued chemotherapy after HBV reactivation and continued antivirus therapy; it is gratifying that the efficacy of antivirus was good and they successfully completed chemotherapy; up to now, they were in good condition. There was no statistical significance for age, sex, stage, IPI, Ki-67, molecular subtype, and chemotherapy regimens between HBV infection group and HBV-free group (Table 1).

### 3.2. MYC and BCL2 Gene Rearrangements

**Figure 1:**
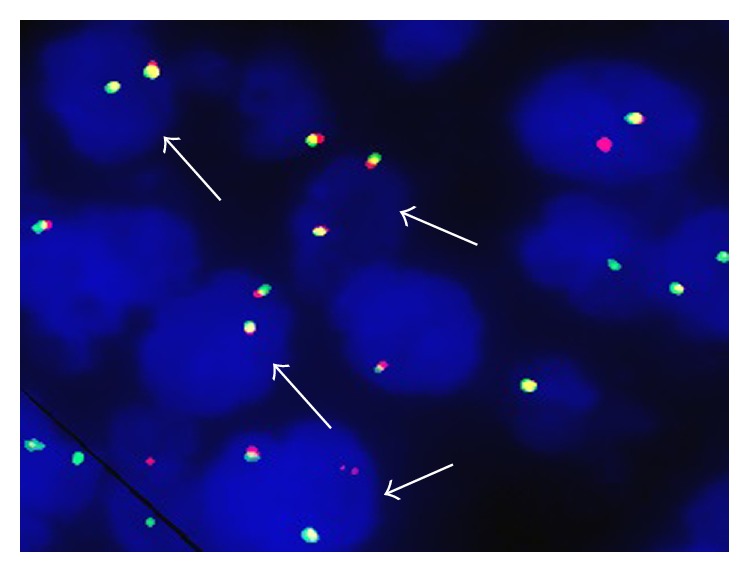
DLBCL patients with normal gene (arrow indicated lymphoma cells without gene rearrangements).

**Figure 2:**
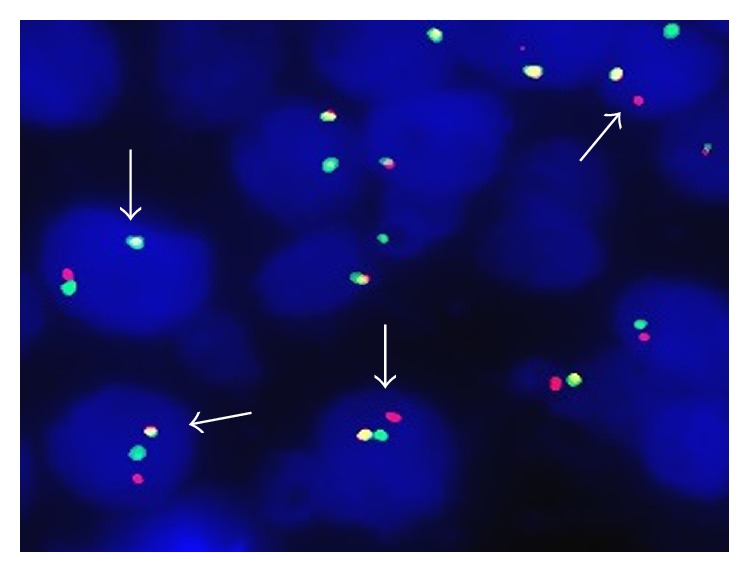
DLBCL patients with gene rearrangements (arrow showed lymphoma cells with gene rearrangements).

*MYC* and* BCL2* gene rearrangements of all patients were analyzed by I-FISH in this study. In HBV infection group, the rate of* MYC* gene rearrangements was 20.0%, which was higher than that (3.9%) in HBV-free group and there was a statistical significance between the two groups (*P* = 0.019) (Figures 1 and 2). The rate of* BCL2* gene rearrangements in HBV infection group was nearly 4-fold higher than that in HBV-free group (23.3% versus 5.9%), and there was also a statistical significance between the two groups (*P* = 0.021) (Table 2). In this study, there was one “double-hit” patient harboring* MYC* and* BCL2* gene rearrangements in HBV infection group.

### 3.3. Survival

**Figure 3:**
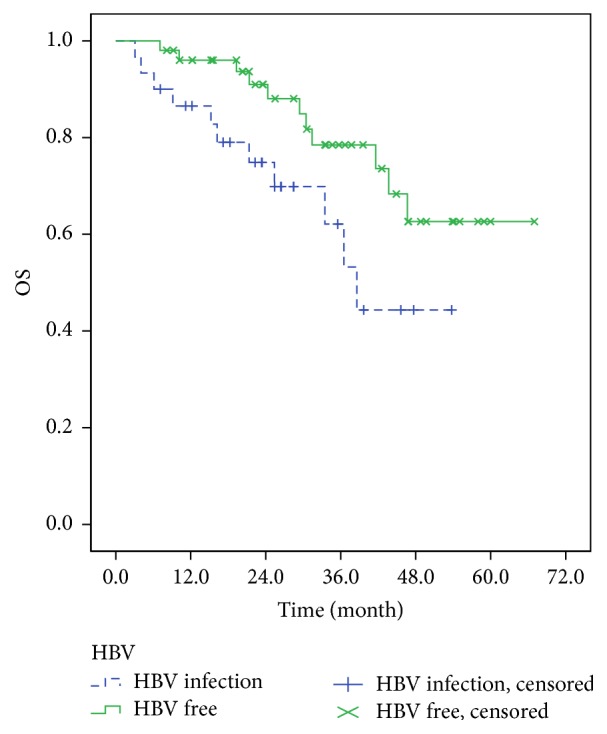
The overall survival of DLBCL patients in HBV infection group and HBV-free group.

**Figure 4:**
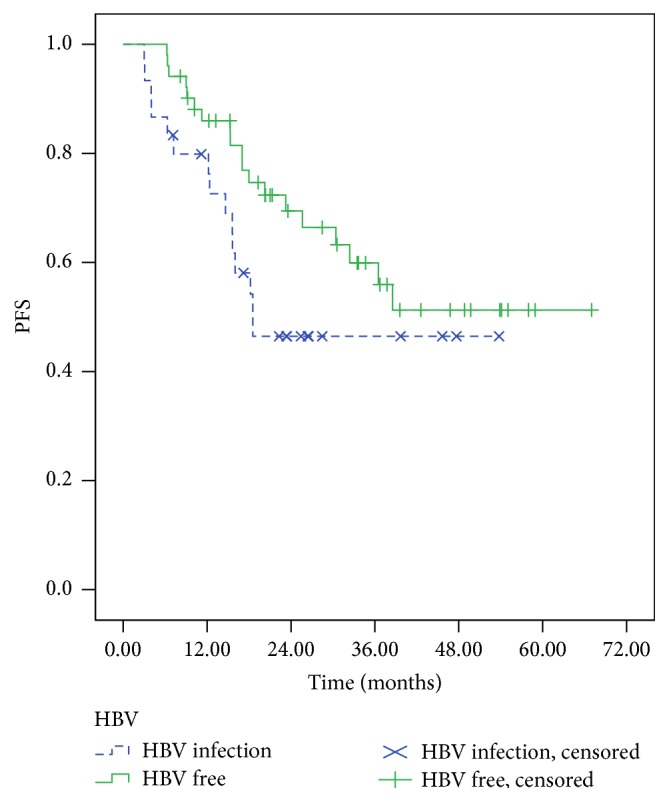
The progression-free survival of patients in HBV infection group and HBV-free group.

As of July 2016, the median follow-up period was 26.4 months (3.0~65.0 months). At the end of follow-up, the median OS of patients in HBV infection group was 38.6 months, while the median OS of patients in HBV-free group was not reached. There was significant difference for OS between the two groups (*P* = 0.042) (Figure 3). Similarly, the median PFS of patients in HBV infection group was worse than that in HBV-free group, 18.5 months and 38.5 months (*P* = 0.118), respectively (Figure 4).

**Figure 5:**
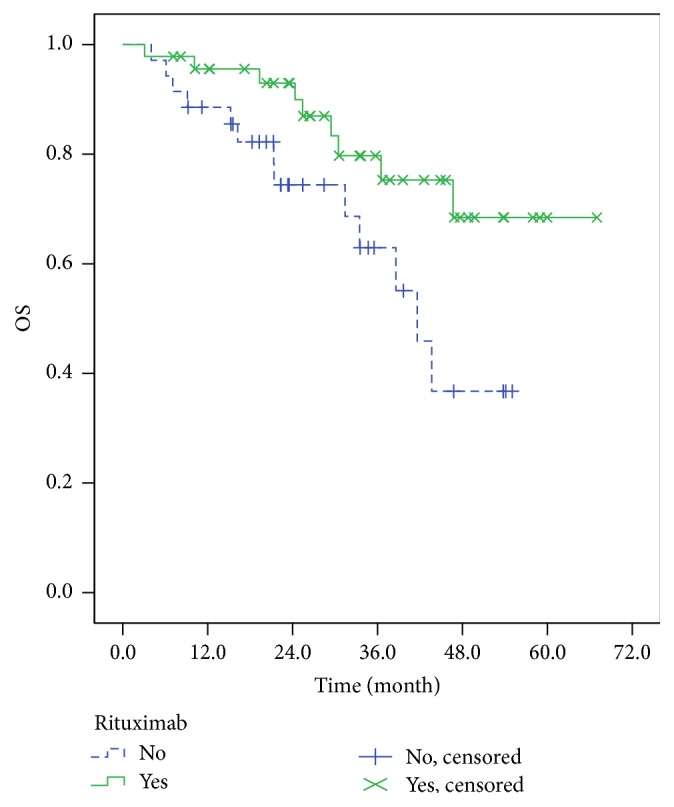
The OS patients between rituximab-containing group and rituximab-free group.

**Figure 6:**
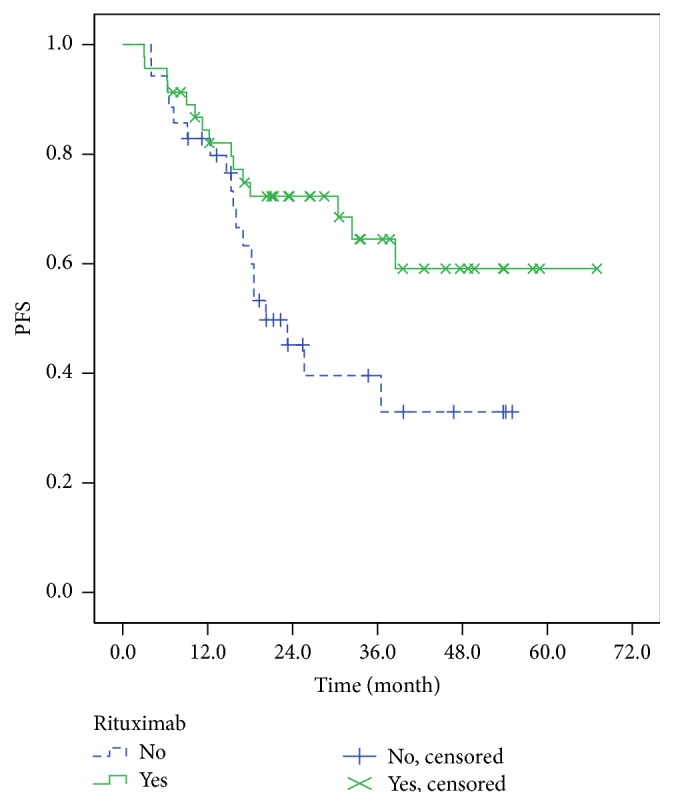
The PFS of patients between rituximab-containing group and rituximab-free group.

We further analyzed OS and PFS of patients between rituximab-containing chemotherapy and rituximab-free chemotherapy, and results indicated that the median OS of patients in HBV infection group was worse than that in HBV-free group, 41.6 months versus not reached (*P* = 0.032), respectively (Figure 5); similarly, the median PFS of patients in HBV infection group was also worse than that in HBV-free group, 20.2 months versus not reached (*P* = 0.042), respectively (Figure 6).

### 3.4. Analysis of Independent Risk Factors

Single variable, including sex, age, clinical stage, IPI, Ki-67, molecular subtype, chemotherapy regimens,* MYC* and* BCL2* gene rearrangements, and HBV status were analyzed by Cox regression analysis in this study, and results revealed that IPI (*P* = 0.003), chemotherapy regimens (*P* = 0.038),* MYC* gene rearrangements (*P* = 0.005), and HBV status (*P* = 0.048) could be clinical adverse prognostic factors for all patients (Table 3); and then multivariable analysis was performed; results showed that IPI (*P* = 0.002), chemotherapy regimens (*P* = 0.017), and* MYC* gene arrangements (*P* = 0.004) were independent adverse prognostic factors for all patients in this study.

## 4. Discussion

In this study, we investigated the prognosis and risk factors of DLBCL patients with HBV infection. The results indicated that (1) the incidence of* MYC* and* BCL2* gene rearrangements in HBV infection group was higher than that in HBV-free group; (2) the median OS and PFS of patients with HBV infection was worse than those with HBV-free; (3) multivariable analysis indicated that IPI, chemotherapy regimens, and* MYC* gene rearrangements were independent prognostic factors for DLBCL patients.

The rate of* MYC* gene rearrangements was 20% in patients with HBV infection, and this result was inconsistent with the previously published articles in which* MYC* gene rearrangements were detected in almost all cases of BL but in less than 10% of the DLBCL patients at diagnosis [13, 14]. For this difference, we believed that HBV infection may remarkably promote the* MYC* gene rearrangements. With regard to the rate of* BCL2* gene rearrangements, previous studies reported that the proportion of* BCL2* gene rearrangements was from 20% to 30% in DLBCL [15]. In this study, the rate of* BCL2* gene rearrangements was 23.3% in patients with HBV infection in the cohort, which was consistent with the reports mentioned above.

In this cohort, the median OS and PFS of DLBCL patients with HBV infection was shorter than those with HBV-free, and then we further explored potential adverse prognostic factors of DLBCL patients, such as sex, age, clinical stage, IPI, Ki-67, molecular subtype, chemotherapy regimens,* MYC* and* BCL2* rearrangements, and HBV status; and multivariable analysis showed that IPI, chemotherapy regimens, and* MYC* gene rearrangements were independent risk factors for DLBCL patients.

IPI is the most common clinical tool used to evaluate the prognosis of DLBCL patients, which includes five prognostic factors with age, Ann Arbor clinical stage, performance status, serum lactate dehydrogenase, and the number of extranodal sites of disease. Based on these factors, DLBCL patients were stratified into four risk categories, namely, low risk, low intermediate risk, high intermediate risk, and high risk. The four risk groups have significantly different five-year OS rates of 73%, 51%, 43%, and 26%, respectively [16]. A study from China explored the poor prognostic factors of patients with DLBCL, and results showed that IPI 3–5 scores affected OS and PFS of DLBCL patients [17]. In this study, the incidence of patients with IPI > 2 in HBV infection group was almost identical to that of HBV-free group; in addition, results from multivariable analysis revealed that IPI was an independent adverse prognostic factor for all patients; therefore, we considered that the poor survival of patients in HBV infection group may be associated with IPI.

Rituximab, an anti-CD20 humanized chimeric monoclonal antibody plus CHOP chemotherapy (cyclophosphamide, doxorubicin, vincristine, and prednisolone) is currently a standard treatment of DLBCL. The application of rituximab obviously increases response rates. What is more, there are also improvements in survival. A 10-year retrospective follow-up analysis results indicated that there were significant differences between R-CHOP and CHOP for OS and PFS for all DLBCL patients [18]. Six-year results of an open-label randomized study of the MabThera International Trial (MInT) Group displayed that rituximab-containing chemotherapy improved long-term outcomes for young DLBCL patients with good prognosis [19]. In this study, the rate of patients who received rituximab-containing chemotherapy in HBV infection group was lower than that in HBV-free group; what is more, we further investigated clinical survival of patients between rituximab-containing group and rituximab-free group, and results showed that the median OS and PFS of patients in rituximab-free group was worse than that in rituximab-containing group; thence, we believed that the poor survival of patients with HBV infection was closely involved in chemotherapy regimens.

*MYC* gene rearrangements were reported to be involved in inferior survival of DLBCL patients. Kojima et al. explored the association between* MYC* rearrangements and the overall survival of DLBCL patients, and multivariable analysis showed that* MYC* rearrangements were independent adverse prognostic factor [20]. Similarly, Aukema et al. also investigated the influence of* MYC* gene rearrangements for the overall survival of DLBCL patients, and the results indicated that patients with* MYC* gene rearrangements had a poor survival compared to the patients without the* MYC* gene rearrangements [21]. Tzankov et al. found that patients with* MYC* rearrangements were more likely to be treatment-resistant (*P* < 0.0001) and had a poor prognosis [22]. Our results indicated that the incidence of* MYC* gene arrangements was high in DLBCL patients with HBV infection, and it was also an independent adverse prognostic factor for those patients. Therefore, we considered that the poor prognosis of DLBCL patients with HBV infection was closely associated with* MYC* gene rearrangements.

The relationship between* BCL2* gene rearrangements and the OS of DLBCL patients remains controversial now. Barrans SL reported that DLBCL patients with* BCL2* gene rearrangements had a decreased survival compared with those without* BCL2* gene rearrangements [23], whereas Kawasaki et al. found that DLBCL patients with* BCL2* gene rearrangements had a better survival than those without* BCL2* gene rearrangements [24]. In present study, although the rate of* BCL2* gene rearrangements was high in DLBCL patients with HBV infection, multivariable analysis showed that* BCL2* gene rearrangements was not an independent adverse prognostic factor. Therefore, we considered that* BCL2* gene rearrangements were not involved with the poor prognosis of DLBCL patients with HBV infection.

The incidence of* MYC* and* BCL2* gene rearrangements in HBV infection group was higher than that in HBV-free group. Some studies found that chronic-phase hepatitis may increase the expression of* c-myc* and* BCL2* gene [25, 26]. According to the existing evidences, we considered that HBV infection may promote* MYC* and* BCL2* gene rearrangements, but the distinct mechanism is still unclear now.

In conclusion,* MYC* and* BCL2* gene rearrangements were common in DLBCL patients with HBV infection. Multivariable analysis revealed that IPI, chemotherapy regimens, and* MYC* gene rearrangements were independent adverse prognostic factors for DLBCL patients. But, as the limited case number in the study, large-scale multicenter clinical studies are needed to verify the results in our study.

## Figures and Tables

**Table 1:** Patient and disease characteristics.

	HBV infection group (*n* = 30)	HBV-free group (*n* = 51)	*P* value
Age			
≥60	9 (30.0%)	20 (39.2%)	0.403
<60	21 (70.0%)	31 (60.8%)	
Sex			
male	18 (60.0%)	27 (52.9%)	0.537
female	12 (40.0%)	24 (47.1%)	
Stage			
I/II	12 (40.0%)	23 (45.1%)	0.655
III/IV	18 (60.0%)	28 (54.9%)	
IPI			
>2	11 (36.7%)	21 (41.2%)	0.688
≤2	19 (63.3%)	30 (58.8%)	
Ki-67			
>80%	10 (33.3%)	9 (17.6%)	0.108
≤80%	20 (66.7%)	42 (82.4%)	
Molecular subtype			
GCB	8 (26.7%)	14 (27.5%)	0.939
Non-GCB	22 (73.3%)	37 (72.5%)	
Rituximab			
Yes	13 (43.3%)	33 (64.7%)	0.061
No	17 (56.7%)	18 (35.3%)	
Antivirus therapy			
Lamivudine	21 (70.0%)	0	NA
Entecavir	9 (30.0%)	0	

NA = no application.

**Table 2:** The rate of MYC and BCL2 gene rearrangements.

Gene rearrangements	HBV infection group (*n* = 30)	HBV-free group (*n* = 51)	*P* value
*MYC*			
Positive	6 (20.0%)	2 (3.9%)	0.019
Negative	24 (80.0%)	49 (96.1%)	
*BCL2*			
Positive	7 (23.3%)	3 (5.9%)	0.021
Negative	23 (76.7%)	48 (94.1%)	

**Table 3:** Results of the Cox regression to evaluate independent risk factors affecting overall survival of all patients in this study.

Characteristics	Univariable analysis			Multivariable analysis		
	HR	95% CI of HR	*P* value	HR	95% CI of HR	*P* value
Sex	1.512	0.613–3.725	0.369			
Age (≥60)	0.540	0.233–1.251	0.151			
Stage (III/IV)	0.410	0.151–1.113	0.080			
IPI (>2)	3.876	1.572–9.524	0.003	4.274	1.721–10.638	0.002
Ki-67 (>80%)	2.036	0.602–6.884	0.252			
Molecular subtype	0.605	0.222–1.645	0.325			
Rituximab	0.404	0.172–0.949	0.038	0.333	0.135–0.820	0.017
MYC positive	4.274	1.553–11.765	0.005	5.556	1.706–18.182	0.004
BCL2 positive	0.507	0.166–1.545	0.232			
HBV status	2.338	1.006–5.432	0.048	1.215	0.487–3.034	0.677

*Note*. HR, hazard ratio; CI, confidence index; IPI, international prognostic index.
